# Measuring Vehicle Profile Size: Lidar-Based System and K-Frame-Based Methodology

**DOI:** 10.3390/s21186206

**Published:** 2021-09-16

**Authors:** Qiang Zhang, Zihao Wang, Jianwen Shao, Libo Weng, Fei Gao

**Affiliations:** 1 College of Computer Science and Technology, Zhejiang University of Technology, Hangzhou 310023, China; zqzjut@126.com (Q.Z.); wenglibo@zjut.edu.cn (L.W.); 2 Institute of Transportation and Acoustical Metrology, Zhejiang Institute of Metrology, Hangzhou 310018, China; wzh330311756@gmail.com (Z.W.); jianwenshao@126.com (J.S.)

**Keywords:** lidar, vehicle profile size measurement, K-frame-based methodology, calibration

## Abstract

At present, light curtain is a widely-used method to measure the vehicle profile size. However, it is sensitive to temperature, humidity, dust and other weather factors. In this paper, a lidar-based system with a K-frame-based algorithm is proposed for measuring vehicle profile size. The system is composed of left lidar, right lidar, front lidar, control box and industry controlling computer. Within the system, a K-frame-based methodology is investigated, which include several probable algorithm combinations. Three groups of experiments are conducted. An optimal algorithm combination, A16, is determined through the first group experiments. In the second group experiments, various types of vehicles are chosen to verify the generality and repeatability of the proposed system and methodology. The third group experiments are implemented to compare with vision-based methods and other lidar-based methods. The experimental results show that the proposed K-frame-based methodology is far more accurate than the comparative methods.

## 1. Introduction

In recent years, researches about intelligent transportation system has been developing rapidly and various research results have been widely applied, which are changing and affecting our life day by day. With the continuous improvement of various performances of automobiles, higher and higher requirements have been put forward for automobile performance detection technology. With the increasing of performance parameters, more and more kinds of testing instruments and equipment are needed and they are developing towards miniaturization, full-automatic and intelligent direction. These instruments and equipment can be used to the vehicle handling performance, safety and reliability, emission environmental protection and other performance indicators for a full range of testing and evaluation.

Since the vehicles are produced according to some standard, many people are not satisfied with the monotonous style, color, or size. So, some vehicles will be modified to satisfy their individual requirements, e.g., adding some parts to enlarge the size to load more materials, etc., which may bring safety hazards like permanent damage to the road, accident and influence on normal driving. To avoid hidden dangers caused by illegal modification, all kinds of vehicles especially trucks need to be regularly measured at the inspection station. Nowadays, several methods such as Coordinate Measuring Machine (CMM) based method [1], light curtain-based method [2], vision-based method [3,4] and lidar-based method [5] were presented to measure the vehicle profile size. There are several limitations about the afore-mentioned methods such complex installation, harsh lighting requirements, sensitive to illuminations, high cost, etc. In this paper, a 2D lidar-based measurement system for vehicle profile size is proposed and a K-frame-based methodology is also investigated. In contrast to most afore-mentioned solutions, the proposed system installs three 2D lidars on fixed brackets to collect point cloud data of vehicles. The point cloud data is used to calculate the profile size of a vehicle through a proposed K-frame-based methodology. The scheme in this paper can better adapt to complex real scenarios with high reliability and measurement accuracy. The main contributions are as follows:

1. A novel solution of lidar-based system for automated measuring vehicle profile size is designed. A method is also investigated to calibrate the parameters of lidar selves and the whole system, which can improve the accuracy and efficiency of system parameter calibration and ensure the accuracy of original point cloud data;
2. An original point cloud data filtering method and a vehicle profile size measurement method are put forward, which can overcome the slight deflection and incline of vehicles and reduce the interference of vibration, smoke and noise in real measuring scenarios;
3. A K-frame-based methodology is proposed to eliminate the measurement error caused by the deviation angle between driving track and central axis.

## 2. Related Work

The CMM-based method [1] can reach a high measurement accuracy while the limitations are obvious such as severe working condition, high cost and time-consuming. The light curtain-based method [2] is a usual method since the advantages are relatively convincing, for instances, simple measurement principle, less requirement for vehicle color and surroundings and so on. Similarly, disadvantages including complex installation process and harsh lighting requirements are also obvious.

Vision-based methods include measurement method based on digital photography and coded mark points [3], binocular stereo-based method [4], structured light and color coding based on method [6], etc. Jia et al. [7] proposed an effective field measurement method for large objects based on a multi-view stereo vision system, the method is effective at measuring the height of a large hot forging. Pu et al. [8] proposed a new method for measuring object size using an ordinary digital camera. Rong et al. [9] proposed a measurement method based on the dual camera vision system and the relative measurement principle and achieved high precision measurement of bayonet size of large automobile brake pads. Zhang et al. [10] proposed a method of sheep body size measurement based on visual image analysis, which can be actually applied in farm environment without disturbing animals. Wang et al. [11] proposed a new portable automatic pig body size measurement system. These methods are of lower costs but depend heavily on good lighting. In addition, vision-based method is of low measurement accuracy and not robust. 

In contrast to afore-mentioned methods, lidar-based method [12] has many advantages, such as higher accuracy, stronger anti-jamming ability, lower environmental requirements and higher reliability. The key of lidar-based method lies in lidar parameter calibration, point cloud data analysis and processing, etc. Many lidar-based methods and systems were developed and applied on object size measurement. Li et al. [12] proposed a non-contact laser scanning based 3D measurement system to obtain the structure of vegetation canopy, which is based on the flying point scanning triangulation. Yin et al. [13] introduced the measurement principle of 3D lidar sensor and analyzed the relationship between projection points, measurement distance and object size. Xu et al. [14] proposed an automatic untouched measurement based on terrestrial 3D lidar (FARO Photon120), which facilitated plane scattered data point convex hull algorithm and slice segmentation and accumulation algorithm to calculate the tree crown projection area. Yang et al. [15] used 3D lidar to obtain point data, analyze point data, obtain plane information, combine plane information with position information and establish 3D model. Wu et al. [16] studied the morphological characteristics of ravines by using 3D lidar. All the above-mentioned methods employed 3D lidar to measure or model a specific object. 3D lidar can rotate scanning plane to realize 3D scanning and acquire the 3D information of environment more quickly and directly, which brings many applications in the field of engineering measurement, complex terrain robot navigation and so on. However, 3D lidar has the disadvantages of complex installation and high cost.

Among lidar-based methods, 2D lidar also can be used to measure or model the object through cooperating with a movable mechanical device or a specific installation method. Gong et al. [17] proposed a 3D ice shape measurement technique through laser light sheet scanning. Yan et al. [18] detected 3D objects with a 2D laser scanning sensor and proposed specially designed algorithm to collect data and construction three-dimensional images. Sanz-Cortiella et al. [19] installed 2D lidar on the retractable bracket to scan the plant and build a 3D model of the plant and measure its size. Rosell et al. [20] employed remote 2D terrestrial lidars to obtain the 3D profile size of tree orchards. Keightley et al. [21] designed a system composed of linear laser sensors and other devices. The system could be moved by a rotating mechanical arm and be used to calculate the biomass of Agronomy. Dias et al. [22] proposed a 3D reconstruction technology based on the real world environment, which was based on the modification of traditional 2D lidar to simulate 3D lidar. Xu et al. [23] developed mechanical devices and motors to rotate the lidar around fixed points, of which three dimensional space data was obtained by single line lidar and 2D data was transformed into three-dimensional data. Li et al. [24] proposed a whole waveform echo decomposition method to improve the ranging accuracy of the whole waveform lidar. Niola et al. [25] showed a robot with two-dimensional lidar that could be used to reverse engineer objects. Fang et al. [26] proposed a real-time low-cost 3D sensing and reconstruction system, which was suitable for autonomous navigation and large-scale environment reconstruction. Choi et al. [27] investigated a sensing model of building structural deformation, which confirmed that the deformation measurement model based on 2D lidar may be a promising alternative. Ringdahlet al. [28] evaluated several existing diameter estimation algorithms using 2D lidar. An enhancement algorithm for compensating beam width and using multiple scans was also developed and evaluated by Bretschneider et al. [29]. Yamada et al. [30] used a 2D lidar to obtain road shape information. According to the road information, the road damage area was detected automatically. Most above-mentioned methods employed single or multi 2D lidars to obtain three-dimensional information of an object, which were usually installed on a movable mechanism and could reduce the equipment cost. However, the reliability and measurement accuracy were also reduced since the moving mechanical mechanism could not ensure a stable measurement system. So, lidar-mechanism-based solution is not suitable for the scene of measuring vehicle profile size since it is more complex and may include smoke, noise and vibration.

## 3. Lidar-Based Measurement System

### 3.1. Structure and Principle

**Figure 1:**
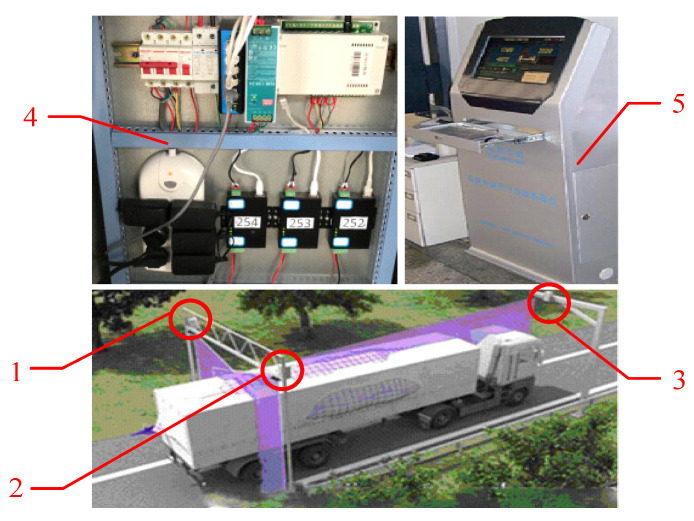
Structure of the proposed system. 1—Left lidar 2—Right lidar 3—Front lidar 4—Control box 5—Industry controlling computer.

As shown in Figure 1, the lidar-based measurement system is composed of left lidar, right lidar, front lidar, control box and industry controlling computer. The left lidar and the right lidar are arranged in the upper left and right corner of the gantry at the entrance of the channel and the front lidar is arranged in the middle of the gantry at the exit of the channel.

**Figure 2:**
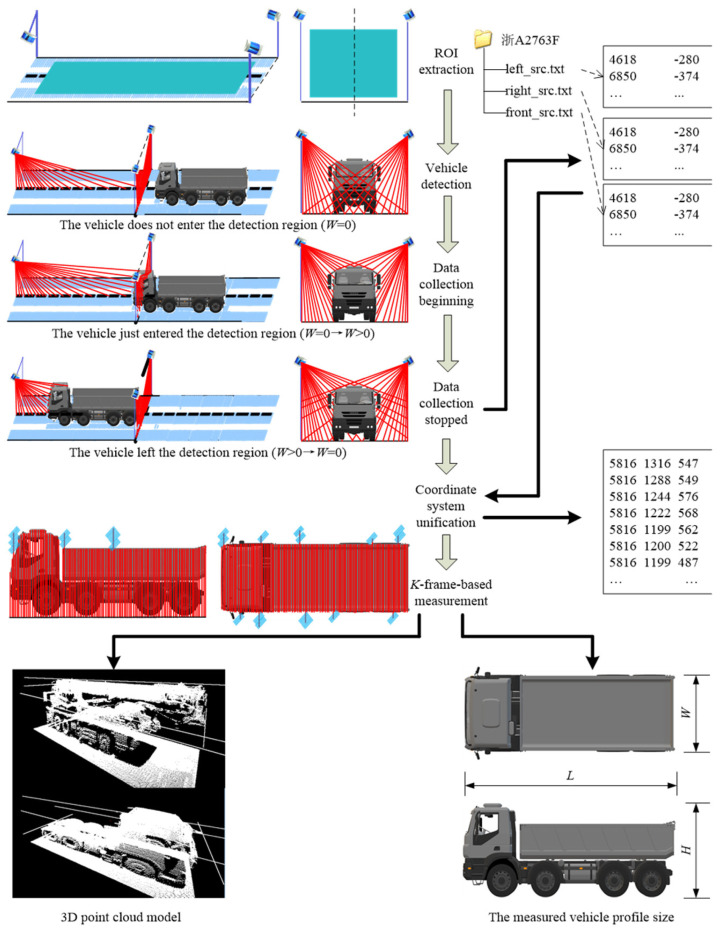
The workflow chart of the measurement process.

The basic principle of the automated measurement system is as follows: when the head of a vehicle is detected to be entering the measurement region, lidars start collecting point cloud data of the vehicle; when the tail of the vehicle is detected to be leaving the measurement region, lidars stop collecting data. The measurement algorithm processes and analyzes the point cloud data and, finally, obtains the vehicle profile size. The workflow chart of the measurement process is depicted as Figure 2.

**Figure 3:**
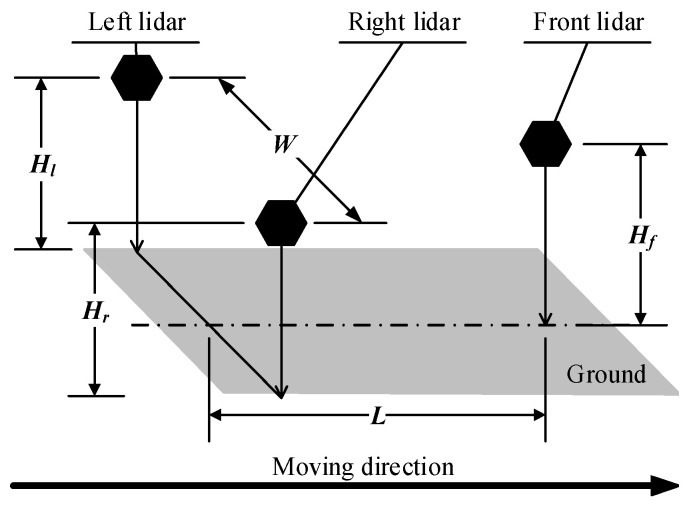
Illustration of lidars installation.

The installation locations of these devices are shown in Figure 3. The point cloud data collected by left, right and front lidars are transformed from TCP/IP protocol data by Ethernet switch in control box and entered into the measurement software installed in the industry controlling computer, which further calculates the length, width and height of the vehicle.

### 3.2. Automated Calibration of Lidars

**Figure 4:**
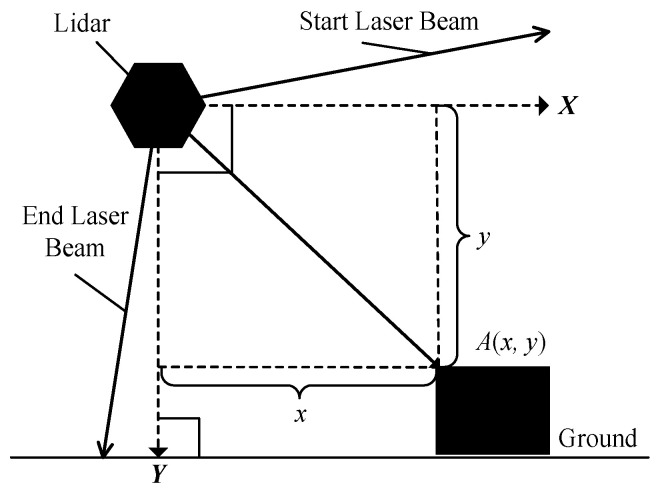
The coordinate system of a lidar and coordinate transformation.

The working principle of lidar is to send a laser beam to an object and then compare the received signal (object echo) reflecting back from the object with the transmitting signal. After proper processing, the distance between the lidar and object can be obtained. To measure the 3D size, the obtained original data needs to be converted into point cloud data in a coordinate system. As shown in Figure 4, a 2D rectangular coordinate system according to each lidar is set up, where the lidar is the origin, vertical downward direction of the lidar is the *Y*-axis and the direction parallel to the ground in the scanning plane of the lidar is the *X*-axis. The coordinate transformation can be obtained according to Equation (1):

(1) $$\{\begin{array}{c} x_{i}=D_{i}sin(\beta_{i})\\ y_{i}=D_{i}cos(\beta_{i}) \end{array}$$

where, *i* represents the serial number of a laser beam in the scanning plane, *D_i_* means the distance between the point on the object that is hit by *i*-th laser beam and the lidar (origin of the coordinate system), *β_i_* is the angle between the *i*-th laser beam and *Y*-axis, (*x_i_*, *y_i_*) is the coordinate point under the coordinate system according to the reflected point on the object as shown in Figure 4.

**Figure 5:**
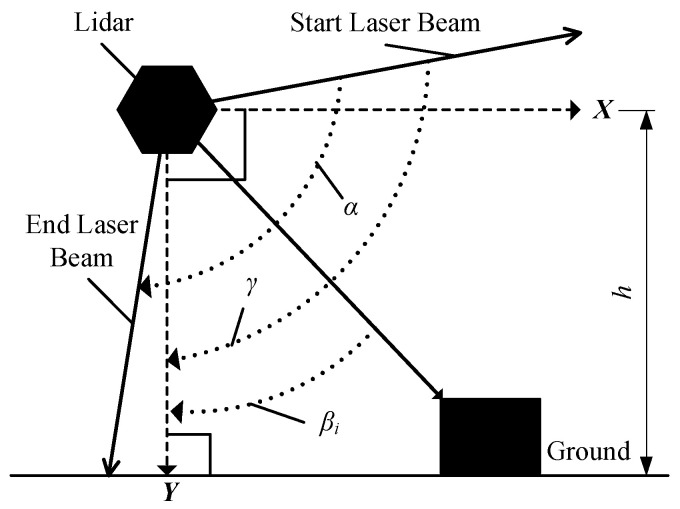
Calibration related angles.

According to the working principle, laser beam scans cyclically from the position of start laser beam to that of the end laser beam with a certain frequency clockwise within a period. As shown in Figure 5, the maximal scanning angle is marked as *α*. According to Equation (1), (*x_i_*, *y_i_*) can be calculated if and only if *β_i_* is known. To calculate *β_i_*, it is necessary to calculate *γ* that is the angle from the position of start laser beam to *Y*-axis, which is called the central angle, then, the installation height of the lidar, *h*, can be calculated.

**Figure 6:**
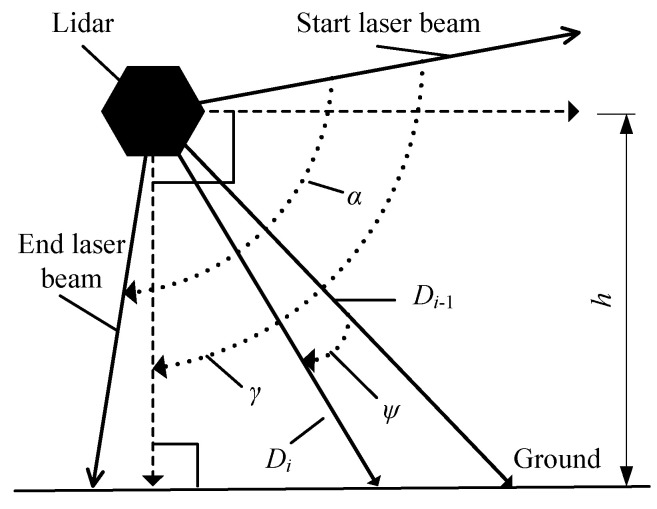
The calibration process.

*β_i_* and *h* are the basic parameters for the automated measurement system of vehicle profile size. How to get the two parameters? When a lidar is working, it continually emits laser beams to the object at regular intervals within a fixed scanning period. Suppose the distance detected by the *i*-th laser beam between the lidar and the reflection point on the object be *D_i_* and a set of the distances be {*D_i_*|*i* = 0, 1, 2, ..., *K* − 1}, *K* is the total number of emitted laser beams within the scanning plane angle *α*. Let the angle of any two adjacent laser beams be *ψ*, which is a constant. Then, *β_i_* and *h* of the lidar can be calculated according to Equation (2). The calibration process is shown in Figure 6.

(2) $$\{\begin{array}{c} h=min{\left\{D_{i}\right\}}_{i=0}^{K-1}\\ \;i^{*}=\underset{i}{argmin}{\left\{D_{i}\right\}}_{i=0}^{K-1}\\ \gamma =i^{*}\times \psi \\ \beta_{i}=\gamma -i\times \psi \end{array}$$

### 3.3. Status Judgment of Vehicle and Frame Data Collection

When the measurement system is started, no matter whether or not there is a vehicle that is moving into the measurement region, the three lidars will keep scanning and continuously transfer the original data frame to the measurement software. Among them, real effective ones are those collected from the time when the vehicle just enters the measurement region to that when it just leaves. So, how to determine when the vehicle enters and leaves the measurement region?

Let point cloud data frames collected by left lidar and right lidar at *t* moment be *LP_t_* = {(*x_i_^l^*^,*t*^, *y_i_^l^*^,*t*^)|*i* = 0, 1, …, *F* − 1} and *RP_t_* = {(*x_i_^r^*^,*t*^, *y_i_^r^*^,*t*^)|*i* = 0, 1, …, *F* − 1}, respectively. *F* presents the number of coordinate points in each frame, (*x_i_^l^*^,*t*^, *y_i_^l^*^,*t*^) represents the converted reflection coordinate point of *i*-th laser beam collected by left lidar, (*x_i_^r^*^,*t*^, *y_i_^r^*^,*t*^) represents the converted reflection coordinate point of *i*-th laser beam collected by right lidar. (*x_i_^l^*^,*t*^, *y_i_^l^*^,*t*^) and (*x_i_^r^*^,*t*^, *y_i_^r^*^,*t*^) are obtained via Equations (1) and (2). *LP_t_* and *RP_t_* are unified into a new coordinate system, of which the right lidar is the origin, horizontal direction is the *X*-axis and vertical downward direction is the *Y*-axis. Then, the new *LP_t_* and *RP_t_* are merged into a uniform set named *P_t_* according to Equation (3). The minimal *X* coordinate value, *x*_min_ and maximal *X* coordinate value, *x*_max_, can be found according to Equation (4). Then, the rule of determining when the vehicle enters and leaves the measurement region is as follows: if (*x*_max_ − *x*_min_) ≥ *w*_0_ is just satisfied, the vehicle is judged to have just entered the measurement region; If (*x*_max_ − *x*_min_) ≥ *w*_0_ is always satisfied after the vehicle is judged to have entered the measurement region, the vehicle is judged to be still in the measurement region; if it changes just from (*x*_max_ − *x*_min_) ≥ *w*_0_ to (*x*_max_ − *x*_min_) *< w*_0_, the vehicle is judged to just have left the measurement region.

(3) $$P_{t}=\{\left(x_{i}^{t},y_{i}^{t}\right)|(\left(x_{i}^{t},y_{i}^{t}\right)\in LP_{t}\;||\;\left(x_{i}^{t},y_{i}^{t}\right)\in RP_{t})\;\&\&\;y_{i}^{t}\geq h_{0}\}$$

(4) $$\{\begin{array}{c} x_{min\;}=\min\{x_{i}^{t}|\left(x_{i}^{t},y_{i}^{t}\right)\in P_{t}\\ x_{max\;}=\max\{x_{i}^{t}|\left(x_{i}^{t},y_{i}^{t}\right)\in P_{t} \end{array}$$

where *h*_0_ represents the vehicle height threshold, *w*_0_ represents the vehicle width threshold.

When the vehicle is judged to have just entered the measurement region, the measurement software begins to record the data frames until the vehicle is judged to just leave the measurement region.

## 4. K-Frame-Based Measurement Methodology

### 4.1. Vehicle Width Measurement

Let the unified point cloud data sets collected by left and right lidar be *LP* = {(*x_ij_*, *y_ij_*)|*i* ∈ [0, *n* − 1], *j* ∈ [0, *F* − 1]} and *RP* = {(*x_kv_*, *y_kv_*)|*k* ∈ [0, *m* − 1], *v* ∈ [0, *F* − 1]}, respectively, *n* represents the quantity of data frames collected by left lidar and *m* represents the quantity of data frames collected by right lidar, (*x_ij_*, *y_ij_*) represents the *j*-th point in the *i*-th frame, (*x_kv_*, *y_kv_*) represents the *v*-th point in the *k*-th frame, *F* represents the quantity of coordinate points in each data frame. Since there may be some noise data due to the interference of dust, smoke, etc., the points near the ground or significantly higher than the vehicle must be preliminarily removed, i.e., those points in *LP* that meet the requirements of *y_ij_* > *h*_1_ or *y_ij_* < *h*_2_ will be removed. Similarly, the points in *RP* that meet the requirements of *y_kv_* > *h*_1_ or *y_kv_* < *h*_2_ are also removed. *h*_1_ represents the upper threshold value in the *Y*-axis direction, which usually represents the ordinate *Y*-axis value near the top of the vehicle. *h*_2_ represents the lower threshold value in the *Y*-axis direction, which usually represents the ordinate *Y*-axis value near the ground. Let the denoised point cloud dataset be *L* and *R*, respectively.

Theoretically, the vehicle width can be obtained easily by subtracting the minimum *X*-axis value *R* from the maximum *X*-axis value in *L*. However, considering that the data collections of left and right lidar are usually not synchronous, it is a problem that which two data frames in *R* and *L* should be chosen for subtraction. In addition, if the single frame subtraction method is adopted, it will bring random error. To overcome the random error caused by the asynchronous collections, a K-frame-based measurement algorithm is proposed. The principle of K-frame-based method is that point cloud data of left and right lidars is sub-grouped according to *K*_1_ and *K*_2_ frames, respectively, namely, each sub-group of point cloud data of left lidar includes *K*_1_ frames and each sub-group of point cloud data of right lidar includes *K*_2_ frames. The algorithm can be represented by Equations (5)–(7).

(5) $$XL_{\Theta }=\underset{i=K_{1}*t,K_{1}*t+1,\cdots ,K_{1}*\left(t+1\right)-1,\;t=0,1,\cdots ,N_{1}-1}{\left\{xl_{t}|xl_{t}=\Theta \{\max\{x_{ij}|j\in \left[0,F-1\right]\}\}\right\}}$$

(6) $$XR_{\Lambda }=\underset{k=K_{2}*t,K_{2}*t+1,\cdots ,K_{2}*\left(t+1\right)-1,\;t=0,1,\cdots ,N_{2}-1}{\left\{xr_{t}|xr_{t}=\Lambda \{\min\{x_{ks}|s\in \left[0,F-1\right]\}\}\right\}}$$

(7) $$W=\Gamma \left(XL_{\Theta },XR_{\Lambda }\right)$$

where *W* represents the final result of vehicle width; *L* is divided into *N*_1_ = *n*/*K*_1_ sub-groups, each sub-group includes *K*_1_ frames; *N*_1_ = *n*/*K*_1_, *K*_1_ is preset; similarly; *R* is divided into *N*_2_ = *m*/*K*_2_ sub-groups, each sub-group includes *K*_2_ frames, *K*_2_ is also preset; *x_ij_* represents the *X* coordinate of *j*-th point in the *i*-th frame in *L*; *Θ* may be a certain operation like max, min, or average, etc.; *xl_t_* represents the result value of the *t*-th sub-group of data frame set in *XL_Θ_* via *Θ* operation; *XL_Θ_* represents the result set of all *N*_1_ sub-groups; *x_ks_* represents the *X* coordinate of *s*-th point in the *k*-th frame in *R*; *Λ* may be a certain operation like max, min, or average, etc.; *xr_t_* represents the result value of the *t*-th sub-group of data frame set in *XR_Λ_* via *Λ* operation; *XR_Λ_* represents the result set of all *N*_2_ sub-groups; *Γ* represents a certain operation like *Θ* and *Λ*; min and max represent the minimum operation and the maximum operation, respectively.

According to the above-mentioned model, different methods can be derived from different *Θ*, *Λ* and *Γ*, as shown in Table 1, *Γ* can be either Equations (8), (9), (10) or (11).

(8) $$\Gamma \left(XL_{\Theta },XR_{\Lambda }\right)=\left|\mathrm{mean}\left(XL_{\Theta }\right)-\mathrm{mean}\left(XR_{\Lambda }\right)\right|$$

(9) $$\Gamma \left(XL_{\Theta },XR_{\Lambda }\right)=\left|\min(XL_{\Theta })-\max(XR_{\Lambda })\right|$$

(10) $$\Gamma \left(XL_{\Theta },XR_{\Lambda }\right)=\max\left\{\left|xr_{t}-xl_{t}\right||t\in \left[0,\min(N_{1},N_{2}\right)]\right\}$$

(11) $$\Gamma \left(XL_{\Theta },XR_{\Lambda }\right)=\mathrm{mean}\left\{\left|xr_{t}-xl_{t}\right||t\in \left[0,\min(N_{1},N_{2}\right)]\right\}$$

In the actual measurement, it is difficult to keep the driving direction completely parallel to the lane axis. In this case, when Equations (8) and (9) are adopted for *Γ*, it will lead to a large error between the measured value of vehicle width and the actual one. So, Equations (10) or (11) is often employed for *Γ* in the actual measurement. The proposed K-frame-based method can effectively reduce the measurement error.

### 4.2. Vehicle Length Measurement

**Figure 7:**
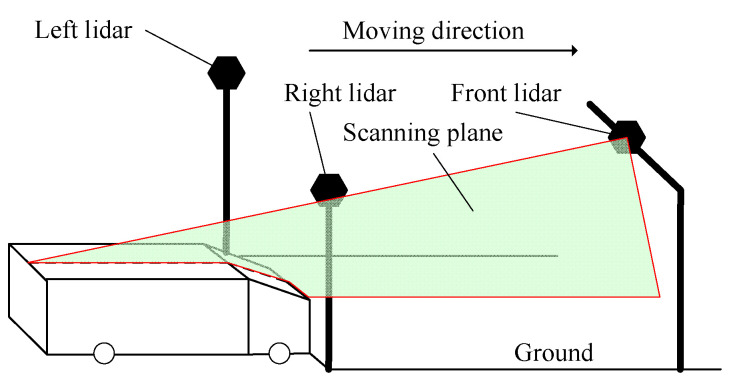
State of *t*_start_.

**Figure 8:**
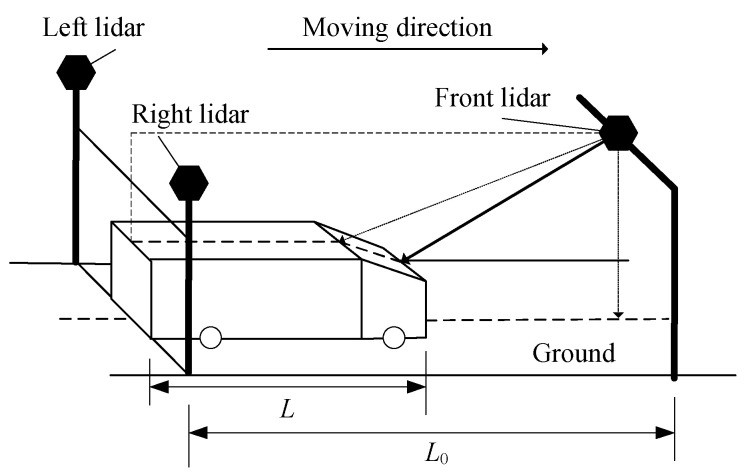
State of *t*_end_.

As shown in Figure 7 and Figure 8, in the vehicle length measurement, the function of left and right lidar is to get the time *t*_start_ when the vehicle enters the measurement region and the time *t*_end_ when the vehicle leaves the measurement region. The length *L*_0_ of the measurement region is already determined when the equipment is installed. At the moment of *t*_start_, the system collects a frame of point cloud data of the front lidar. After preliminary filtering, the *X* coordinate set $\left\{x_{t}^{\mathrm{start}}\right\}$ of point cloud data is obtained. At the moment of *t*_end_, the system also collects a frame of point cloud data of the front lidar. Similarly, after preliminary filtering, the point cloud data *X* coordinate set $\left\{x_{t}^{\mathrm{end}}\right\}$ is obtained. Then, the vehicle length *L* can be calculated according to Equation (12).

(12) $$L=\min\{x_{t}^{\mathrm{start}}\}-\min\{x_{t}^{\mathrm{end}}\}$$

### 4.3. Vehicle Height Measurement

**Figure 9:**
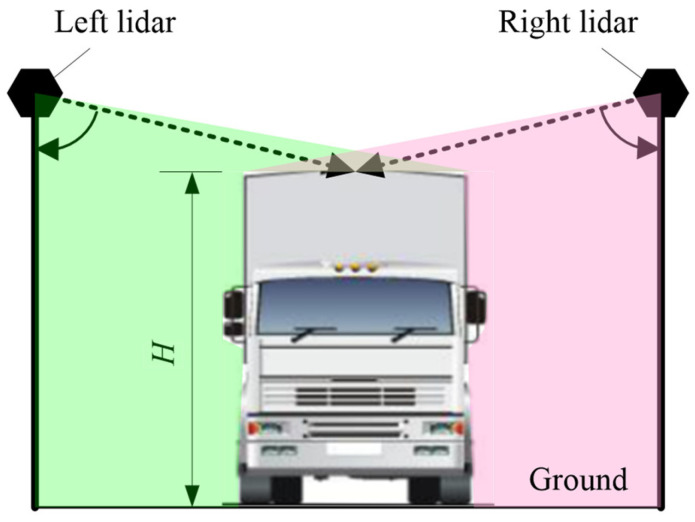
Illustration of how to measure vehicle height.

The algorithm of vehicle height measurement is similar to that of vehicle width measurement. Theoretically, the left or right lidar can measure the vehicle height separately, as shown in Figure 9; however, this may lead to a large error. To eliminate the measurement error, the point cloud data of both the two lidars are still fully fused and an algorithm similar to that of vehicle width measurement is advised. So, the vehicle height can be calculated according to Equation (13).

(13) $$H=max\underset{t=0,1,\cdots ,\min\left(N_{1},N_{2}\right)-1}{\left\{h_{t}|h_{t}=\max\left(YL_{\theta },YR_{\Lambda }\right)\right\}}$$

where the calculation methods of *YL_Θ_* and *YR_Λ_* are similar to *XL_Θ_* and *XR_Λ_*.

## 5. Error Analysis

### 5.1. Calibration Error

**Figure 10:**
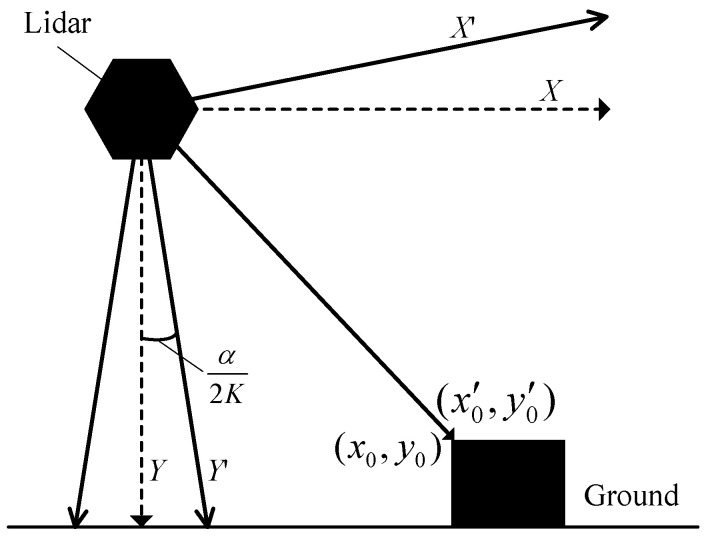
Diagram of maximum calibration error.

**Figure 11:**
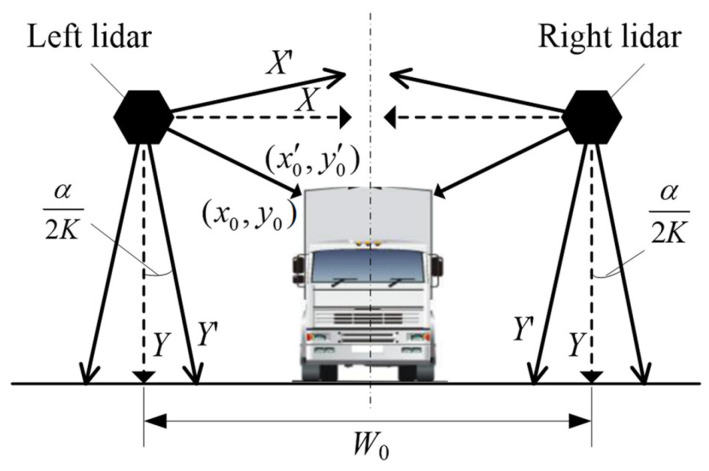
The influence of calibration error on vehicle width measurement.

Suppose the maximum angle of the lidar scanning plane be *α* and total number of laser beams of each scanning cycle be *K*. Due to the limited number of laser beams, sometimes it is impossible to find a laser beam that coincides with the vertical direction as the *Y*-axis. In this case, the closest laser beam is chosen as the *Y*-axis, which absolutely will lead error. As shown in Figure 10, when the vertical line from lidar to ground is exactly in the middle of two laser beams, calibration error will be maximum. Under the situation, the angle value between the closest laser beam and the vertical line is *α/*2*K*. The influence of the calibration error on vehicle width measurement error is shown in Figure 11. The coordinate conversion relationship between the actually established coordinate system and the standard one with the vertical line as the *Y*-axis is shown in Equation (14).

(14) $$\{\begin{array}{l} x_{0}^{\prime }=x_{0}\cos(\alpha /2n)+y_{0}\sin(\alpha /2n)\\ y_{0}^{\prime }=-x_{0}\sin(\alpha /2n)+y_{0}\cos(\alpha /2n) \end{array}$$

If other factors that may cause errors are excluded and the vehicle drives strictly along with the middle of the lane and the horizontal width of the left and right lidars is *W*_0_, the theoretical vehicle width is calculated according to Equation (15). In consideration of calibration error, the actual measured value is shown in Equation (16). Then, the relative measurement error of vehicle width caused by calibration error is according to Equation (17).

(15) $$W=W_{0}-2x_{0}$$

(16) $$W^{\prime }=W_{0}-2\left[x_{0}\cos(\alpha /2K\right)+y_{0}\sin(\alpha /2K)]$$

(17) $$\delta =\left|\frac{W-W^{\prime }}{W}\right|=\frac{2\left[x_{0}\cos(\alpha /2K\right)+y_{0}\sin(\alpha /2K)]-2x_{0}}{W_{0}-2x_{0}}$$

To eliminate the measurement error caused by calibration, a calibration object whose profile size is known is used a fine-tuning strategy is facilitated as follows:

1. a calibration object, whose profile size is known, is measured by the proposed system;
2. the lidars are fine-tuned according to difference value between the measured value and actual value of the calibration object;
3. step 1 and 2 are repeated until the measured value is equal to the actual value.

### 5.2. Deviation Error of Vehicle Moving

**Figure 12:**
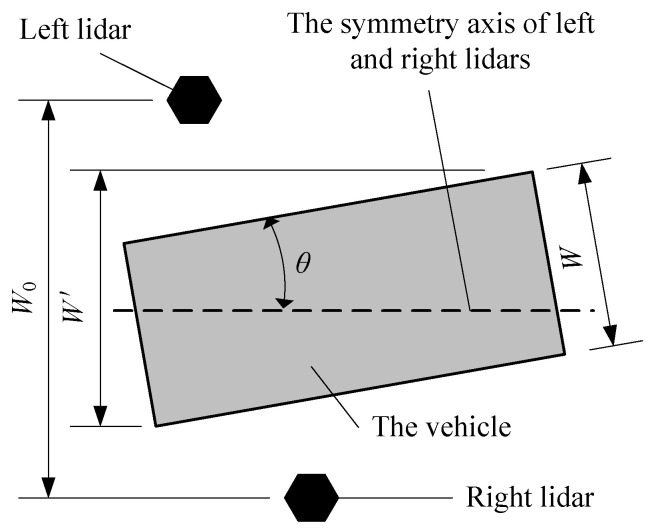
Influence of vehicle moving deviation error on vehicle width measurement.

As shown in Figure 12, in the basic algorithm combination of Table 1, if Equation (8) or Equation (9) is adopted for Γ and there is deflection angle between the track and the central axis when the vehicle is moving forward, the error will be relatively large. Suppose the angle between the vehicle driving direction and the central lane axis be *θ*, the actual value of the vehicle width be *W*, the measured value be *W*^′^ and the actual value of the vehicle length be *L*, the relative error of the vehicle width caused by the vehicle moving deviation is shown in Equation (18).

(18) $$\delta =\left|\frac{W-W^{\prime }}{W}\right|=\left|\frac{\left[W+L\tan(\theta \right)]\cos(\theta )-W}{W}\right|$$

Then, expanding Equation (18) can be expanded to Equation (19).

(19) $$\delta =\left|\cos(\theta )+\frac{L}{W}\sin(\theta )-1\right|$$

Usually, as for trucks, *W* is limited to 2.5 m, *L* is limited to 18 m. If the measurement error *δ* < 1% is required, *θ* < 0.08° must be satisfied; if *L* is limited to 13 m, *θ* < 0.12° must be satisfied, which means the high requirement for vehicle moving trajectory. Thus, a K-frame-based method is proposed, which can eliminate the measurement error caused by the deviation angle between the vehicle and the central axis and overcome the synchronization requirements of the left and right lidar through filtering noise data.

## 6. Experiments

### 6.1. Determination of the Optimal Algorithm

**Figure 13:**
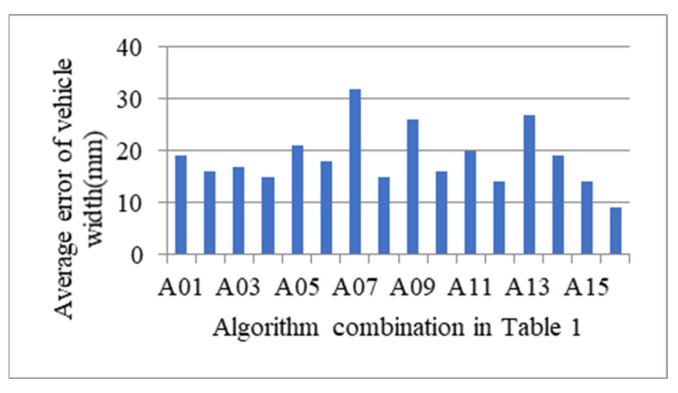
Average error of vehicle width according to algorithm combination in Table 1.

**Figure 14:**
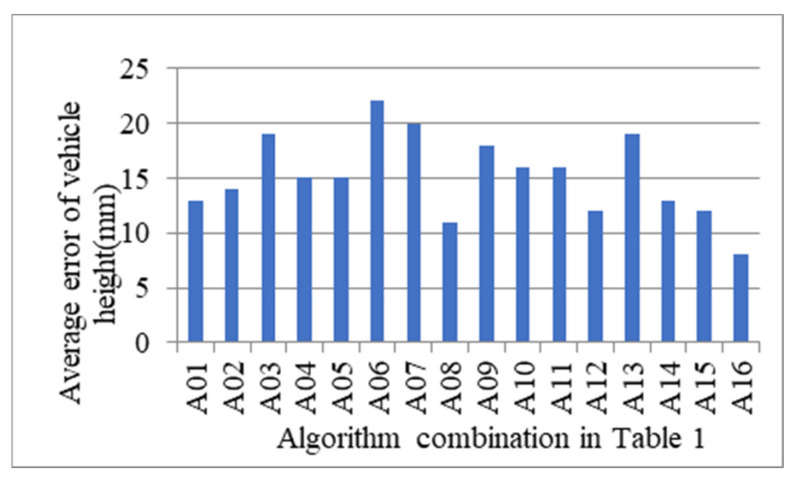
Average error of vehicle height according to algorithm combination in Table 1.

Different combinations according to Table 1 are tested for about 800 vehicles to determine the exact measure algorithm that has the minimal measurement error profile size, where there are 102 vans, 36 barn trucks, 59 fence trucks, 20 crane trucks, 150 semi-trailer tractors and 77 buses. Average errors of vehicle width and height according to algorithm combination in Table 1 are shown in Figure 13 and Figure 14, respectively. According to the experimental results, it can be found that the combination A16 in Table 1, i.e., *Θ* chooses mean method, *Λ* chooses mean method, *Γ* chooses Equation (11), can reach the minimal average error.

**Figure 15:**
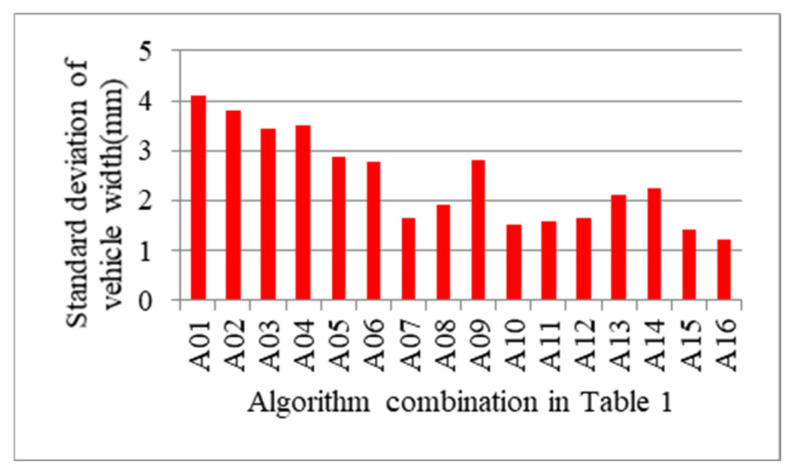
Standard deviation of vehicle width according to algorithm combination in Table 1.

**Figure 16:**
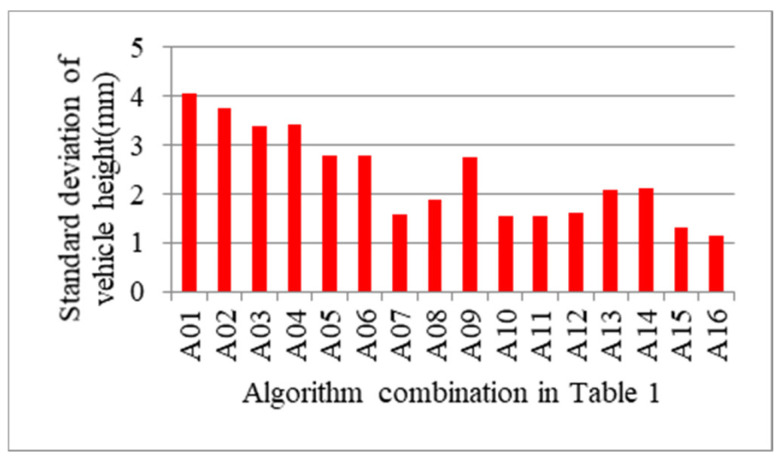
Standard deviation of vehicle height according to algorithm combination in Table 1.

However, decision about which algorithm combination should be chosen can’t be made just according to the average error. Considering that the number of samples of different types of vehicles in the experiment varies greatly, it is very likely that the combination with smaller average error may have smaller error only when measuring the vehicle type with larger number of samples and the error may be too large when measuring the vehicle type with smaller number of samples. Then, the standard deviation of vehicle width and height is also calculated and is depicted in Figure 15 and Figure 16, respectively. The experiment results of the standard deviation show that A16 still reaches the minimal value. On the whole, A16 is an algorithm combination with smaller error and stronger applicability compared with other algorithms, which is determined to be the exact algorithm for measuring the vehicle profile size.

### 6.2. Experiments of the Optimal Algorithm

Five specific vehicles from the above-mentioned five categories are chosen for the experiments to verify the applicability and repeatability of A16, which are semi-trailer tractor, van, barn truck, fence truck, crane truck and bus. The profile size of all the five vehicles is measured ten times and compared with the ground truth. The experimental results, i.e., profile size, max error, relative error and repeatability error, are shown in the Table 2.

**Figure 17:**
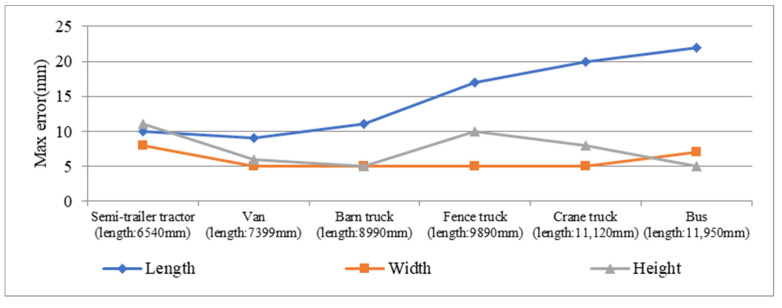
Max error of profile size of the five vehicles.

As shown in Figure 17, max errors of the experimental results are depicted as a line chart in ascending order of vehicle length. The measurement time increases according to the increasing of the vehicle length, where it is supposed that the vehicles move with similar speed. Then, errors caused slight deflection and moving deviation from central axis of the lane will be accumulated. Since it is completely different from the width and height measurement, K-frame-based method is not employed in the length measurement. In Figure 17, it can be clearly found that the max error of length also increases with the increasing of measurement time while that of width and height does not increase with the increasing of measurement time, which further proves that K-frame-based method can eliminate the error caused by moving deviation from central axis of the lane to a certain extent.

**Figure 18:**
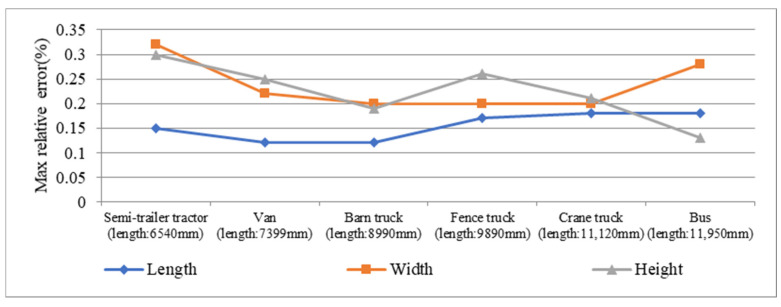
Max relative error of profile size of the five vehicles.

Although Figure 17 shows that the max error of length will increase with the increasing of measurement time, it also can be found that the max relative error of length does not change significantly with the increasing of vehicle length. In fact, as shown in Figure 18, the max relative error of length fluctuates in a small range just similar to the max error of width and height, which shows that the proposed measurement method is of high applicability after facilitating the optimal algorithm combination A16.

**Figure 19:**
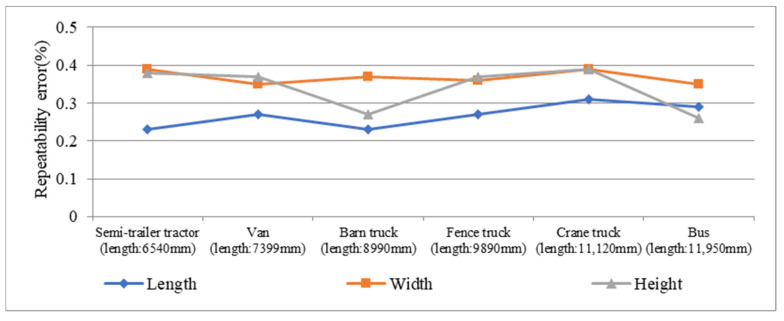
Repeatability error of profile size of the five vehicles.

In terms of repeatability, it can be seen from Figure 19 that the repeatability error is confined to a small range and there is no repeatability error higher than 0.5%, which shows the proposed measurement is of good repeatability.

### 6.3. Comparison Experiments

Several comparison experiments with Li et al. [31], Robert et al. [32] and Robert et al. [33] are conducted to prove the measurement accuracy of the proposed method, where Li et al. [31] investigated a monocular-vision-based method of vehicle 3D size measurement, Robert et al. [32] designed a scheme to measure the length of moving vehicles by employing stereoscopic video analysis technology, Robert et al. [33] proposed a new method to estimate vehicle size based on active appearance model (AAM) and stereoscopic video analysis. Although most of the experimental measurement objects are passenger vehicles with only a few large vehicles, the above-mentioned three methods are still used in our comparison experiments since they behave well in the measurement of vehicle profile size. The experimental results are shown in Table 3. It can be found that the proposed method is far more accurate than the three methods. Since the principle of the proposed method is completely different from those of the three methods, the purpose of this comparison is to verify the high measurement accuracy in the similar scene.

To further prove how good the K-frame-based method with the algorithm combination A16 is, additional comparison experiments with Xu et al. [34] and Xu et al. [35] are implemented. Xu et al. [34] developed a vehicle size measurement method based on monocular vision, Xu et al. [35] proposed a method of vehicle 3D dimension measurement based on laser ranging is proposed and developed a vehicle 3D dimension measurement system. Although only the experiment of dump truck is carried out [34], the application scene of is similar to that of the system in this paper. The hardware and installation scheme in [35] are very similar to ours. Those are why they are chosen for the comparison. Table 4 shows the experiment results. From Table 4, it can be found that in the similar application scenarios, the accuracy of the proposed method is higher than [34] in the similar application scenarios, which proves the correctness of lidar-based measurement equipment. According to the comparison experimental results with [35], it can be seen that the K-frame-based algorithm and the selected optimal algorithm combination A16 can greatly improve the measurement accuracy and prove the significance of K-frame-based algorithm and the selected optimal combination strategy.

Overall, according to the above-mentioned experimental results, the proposed scheme and system has a high measurement accuracy in the application scenarios. Especially, the K-frame-based algorithm and the selected optimal algorithm combination A16 play important roles in improving the measurement accuracy in the system.

## 7. Conclusions

In this paper, a complete solution of lidar-based automated measurement system for vehicle profile size is developed and a method including lidar system calibration, vehicle status determination, original data processing and profile size calculation is proposed, which greatly reduces the influence of various interference factors on measurement accuracy. Especially, a K-frame-based algorithm is investigated can eliminate the measurement error caused by the deviation angle between the vehicle and the central axis when the vehicle is moving forward, at the same time, it can overcome the synchronization requirements of the left and right lidar and filter noise data. The experimental results show that, the proposed method makes a great improvement on the measurement accuracy with similar equipment under similar conditions. At the same time, the K-frame-based algorithm is not limited by hardware devices, which has a certain generality. How to facilitate the algorithm in light curtain and machine vision measurement systems is part of our ongoing work, which may reduce the influence of various interference factors on measurement accuracy and reduce measurement error.

## Figures and Tables

**Table 1:** combination of *Θ*, *Λ* and *Γ*.

No.	*Θ*	*Λ*	*Γ*	Remarks
A01	min	max	Equation (8)	All point cloud data in *L* and *R*
A02	mean	mean	Equation (8)	
A03	min	max	Equation (9)	
A04	mean	mean	Equation (9)	
A05	min	max	Equation (10)	
A06	mean	mean	Equation (10)	
A07	min	max	Equation (11)	
A08	mean	mean	Equation (11)	
A09	min	max	Equation (8)	Remove part of the front and back point cloud frames in *L* and *R*
A10	mean	mean	Equation (8)	
A11	min	max	Equation (9)	
A12	mean	mean	Equation (9)	
A13	min	max	Equation (10)	
A14	mean	mean	Equation (10)	
A15	min	max	Equation (11)	
A16	mean	mean	Equation (11)	

**Table 2:** Experimental results of generality and repeatability of A16.

Type	Size	Ground Truth	Ten Measurements (mm)										Mean (mm)	ME (mm)	MRE (mm)	RE (%)
			1st	2nd	3rd	4th	5th	6th	7th	8th	9th	10th				
Semi-trailer tractor	*L*	6540	6542	6535	6545	6540	6537	6534	6540	6541	6535	6530	6538	−10	−0.15	0.23
	*W*	2535	2533	2540	2535	2536	2537	2533	2543	2540	2535	2533	2537	8	0.32	0.39
	*H*	3710	3711	3707	3700	3713	3710	3702	3699	3709	3710	3711	3707	−11	−0.30	0.38
Van	*L*	7399	7408	7395	7410	7390	7397	7399	7401	7405	7401	7400	7401	9	0.12	0.27
	*W*	2308	2310	2307	2306	2311	2311	2305	2310	2308	2305	2313	2309	5	0.22	0.35
	*H*	2413	2411	2412	2416	2407	2410	2411	2416	2413	2415	2409	2412	−6	−0.25	0.37
Barn truck	*L*	8990	8979	8992	8995	8988	8983	9000	8994	8985	8980	8991	8989	−11	−0.12	0.23
	*W*	2465	2469	2470	2461	2465	2469	2463	2463	2470	2470	2464	2466	5	0.20	0.37
	*H*	2610	2609	2612	2607	2606	2609	2606	2611	2610	2605	2610	2609	−5	−0.19	0.27
Fence truck	*L*	9890	9901	9887	9907	9883	9889	9903	9892	9890	9905	9880	9894	17	0.17	0.27
	*W*	2485	2488	2487	2485	2480	2488	2486	2483	2486	2485	2489	2486	−5	−0.20	0.36
	*H*	3830	3828	3834	3830	3828	3820	3834	3832	3829	3833	3830	3830	−10	−0.26	0.37
Crane truck	*L*	11,120	11,110	11,100	11,126	11,130	11,135	11,113	11,120	11,129	11,108	11,105	11,118	−20	−0.18	0.31
	*W*	2548	2543	2547	2548	2550	2547	2553	2543	2548	2545	2550	2547	5	0.20	0.39
	*H*	3850	3858	3851	3844	3853	3850	3849	3843	3850	3855	3843	3850	8	0.21	0.39
Bus	*L*	11,950	11,963	11,960	11,931	11,940	11,928	11,950	11,943	11,935	11,959	11,928	11,944	−22	−0.18	0.29
	*W*	2540	2538	2537	2542	2540	2533	2540	2542	2541	2537	2538	2539	−7	−0.28	0.35
	*H*	3840	3839	3836	3843	3845	3835	3840	3842	3835	3840	3837	3839	5	0.13	0.26

ME = Max Error; MRE = Max Relative Error; RE = Repeatability Error. The Mean is the average of all measurement results. The Max Error is the biggest error in all measurement results. The Max Relative Error is (the Max Error/the Ground truth) × 100. The Repeatability Error is ((the maximum measurement − the minimum measurement)/the Mean measurement) × 100.

**Table 3:** The experimental results of our measurement method and the three reference comparison methods.

Method	Max Relative Error (%)		
	Length	Width	Height
Li et al. [31]	8.86	6.59	3.90
Robert et al. [32]	47	/	/
Robert et al. [33]	10.69	19.28	18.42
Ours	0.18	0.28	0.30

**Table 4:** The experimental results of our measurement method and the to main comparison methods.

Method	Max Relative Error (%)		
	Length	Width	Height
Xu et al. [34]	1.70	5.30	3.60
Xu et al. [35]	9.3		
Ours	0.18	0.28	0.30

## Data Availability

Further information about the datasets used in the present work are available at the link https://www.dropbox.com/s/xnxf1nqe7psw672/ZPVehicle.rar/ (accessed on 19 December 2019).
